# The Population and Evolutionary Dynamics of Homologous Gene Recombination in Bacteria

**DOI:** 10.1371/journal.pgen.1000601

**Published:** 2009-08-14

**Authors:** Bruce R. Levin, Omar E. Cornejo

**Affiliations:** Department of Biology, Emory University, Atlanta, Georgia, United States of America; University of Toronto, Canada

## Abstract

In bacteria, recombination is a rare event, not a part of the reproductive process. Nevertheless, recombination—broadly defined to include the acquisition of genes from external sources, i.e., horizontal gene transfer (HGT)—plays a central role as a source of variation for adaptive evolution in many species of bacteria. Much of niche expansion, resistance to antibiotics and other environmental stresses, virulence, and other characteristics that make bacteria interesting and problematic, is achieved through the expression of genes and genetic elements obtained from other populations of bacteria of the same and different species, as well as from eukaryotes and archaea. While recombination of homologous genes among members of the same species has played a central role in the development of the genetics and molecular biology of bacteria, the contribution of homologous gene recombination (HGR) to bacterial evolution is not at all clear. Also, not so clear are the selective pressures responsible for the evolution and maintenance of transformation, the only bacteria-encoded form of HGR. Using a semi-stochastic simulation of mutation, recombination, and selection within bacterial populations and competition between populations, we explore (1) the contribution of HGR to the rate of adaptive evolution in these populations and (2) the conditions under which HGR will provide a bacterial population a selective advantage over non-recombining or more slowly recombining populations. The results of our simulation indicate that, under broad conditions: (1) HGR occurring at rates in the range anticipated for bacteria like *Streptococcus pneumoniae*, *Escherichia coli*, *Haemophilus influenzae*, and *Bacillus subtilis* will accelerate the rate at which a population adapts to environmental conditions; (2) once established in a population, selection for this capacity to increase rates of adaptive evolution can maintain bacteria-encoded mechanisms of recombination and prevent invasion of non-recombining populations, even when recombination engenders a modest fitness cost; and (3) because of the density- and frequency-dependent nature of HGR in bacteria, this capacity to increase rates of adaptive evolution is not sufficient as a selective force to provide a recombining population a selective advantage when it is rare. Under realistic conditions, homologous gene recombination will increase the rate of adaptive evolution in bacterial populations and, once established, selection for higher rates of evolution will promote the maintenance of bacteria-encoded mechanisms for HGR. On the other hand, increasing rates of adaptive evolution by HGR is unlikely to be the sole or even a dominant selective pressure responsible for the original evolution of transformation.

## Author Summary

For many species of bacteria, recombination in the form of the acquisition and expression of genes and genetic elements acquired from other bacteria, eukaryotes, and archaea, HGT is an important source of variation for adaptive evolution. Not so clear is the contribution of recombination of homologous genes to adaptive evolution and as a selective pressure for the evolution and maintenance of HGT. Using computer simulations, we explore the role of HGR to adaptive evolution and selection for the evolution and maintenance of HGT. We demonstrate that under realistic conditions by shuffling genes within a bacterial population, HGR will increase its rate of adaptive evolution. Once established, this capacity to increase the rate of adaptive evolution can serve as a selective force for the maintenance of HGT. On the other hand, HGR cannot provide an advantage to a population when its density is low or when the recombining population is rare relative to non-recombining competitors. Thus, we postulate that it is unlikely that the only bacteria—rather than plasmid (or phage)—determined mechanism of HGR, transformation, evolved in response to selection for higher rates of evolution by gene shuffling.

## Introduction

Recombination in the form of the receipt and incorporation of genes and genetic elements from other strains and species of bacteria [1] as well as archaea and eukaryotes [2],[3],[4],[5],[6],[7] plays a prominent role as a source of variation for the adaptive evolution of many species of bacteria [8],[9],[10],[11],[12],[13],[14],[15],[16],[17],[18]. Because of this ability to acquire genes and genetic elements from other organisms, horizontal gene transfer (HGT), the pace of adaptive evolution in bacteria need not be limited by the standing genetic variation within a population or the slow rate by which adaptive genes are generated by recurrent mutation. Through single HGT events bacteria can obtain chromosomal genes and gene clusters (islands) as well as plasmids, transposons and prophage bearing genes that have successfully traversed the gauntlet of natural selection in some other population of their own or other species. In this way, bacteria can expand their ecological niches; colonize new habitats and hosts, metabolize new energy sources, synthesize essential nutrients, survive toxic agents like antibiotics, and alas, increase their virulence to human and other hosts.

Less clear are the ecological and evolutionary consequences of more mundane HGT events, such as homologous gene recombination (HGR) among members of the same population. In accord with classical population genetic theory, meiotic recombination of can increase the rate at which populations adapt to new environments by assembling in single organisms combinations of adaptive mutations occurring in different members of their population and by reducing the rate at which populations accumulate deleterious mutations, (“Muller's Ratchet”). For superb reviews of this classical theory and some of its more recent extensions see [19],[20],[21].

There are good theoretical reasons to anticipate that recombination among chromosomal genes already present and those generated by mutation within a bacterial population can augment its rate of evolution in a variety of ecological situations [22]. There is also experimental evidence in support of this prediction. Transformation occurs at measurable rates in *B. subtilis* maintained in a more or less natural setting and the resulting HGR appears to promote adaptive evolution in these populations [23]. Two recent experimental studies comparing rates of evolution among recombining and non-recombining populations provide direct evidence that capacity for F-plasmid mediated recombination in *E. coli* [24], and transformation-mediated recombination in *Helicobacter pylori* [25], can increase the rate at which these bacteria adapt to culture conditions. However, there is also evidence from studies with experimental populations of *E. coli* [26] and *Acinetobacter baylyi* [27] indicating that there are conditions where there are no differences in the rates at which recombining and non-recombining populations adapt to environmental conditions.

On first consideration, it would seem that if recombination increases the rate at which populations adapt to their environment, the capacity for shuffling homologous genes within a population would provide an advantage to the recombining strain when competing with populations without this capacity. Not so clear are the *conditions* under which selection will favor recombining populations in this way. When will a recombining population prevail over non- or more slowly- recombining populations, and do so in the face of fitness costs associated with the capacity for recombination?

Here, we present the results of a study using computer simulations of mutation, recombination, selection and inter-population competition to explore the conditions under which: i) recombination augments rates of evolution in bacterial populations and, ii) when the capacity for HGR will be favored in competition with non-recombining populations. We demonstrate that under broad conditions, HGR occurring at rates in a range estimated for *E. coli*, *H. influenza*, *S. pneumoniae*,, and *B. subtilis* can increase the rate of adaptive evolution in bacterial populations. We show that this capacity for increasing rates of evolution by shuffling chromosomal genes can provide a recombining population a selective advantage in competition with populations without this capacity even when the recombining population has a lower intrinsic fitness. On the other hand, we also demonstrate that because the rate of recombination in bacteria depends on the density of the recombining population, the conditions under which recombination can provide a population a selective advantage in competition with non-recombining populations are restricted to when the recombining population is relatively common and the total population density is high. Even in the absence of a fitness cost, when the recombining population is rare, it will not be favored despite its ability to acquire genes from the dominant non-recombining population. We discuss the implications of these simulation results to the role of recombination in the adaptive evolution of bacteria and the evolution and maintenance of different mechanisms for homologous gene recombination in bacteria.

## Methods

### Semi-Stochastic Simulations of Mutation, Selection, and Recombination in a Bacterial Population

#### Single population simulation

**Figure 1:**
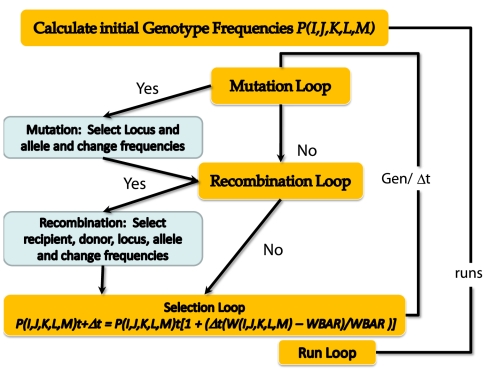
Five-locus, three allele simulation of mutation, recombination, selection and a bacterial population.

In this simulation we consider a population of bacteria in mass (liquid) culture and five loci each with three alleles designated, 1, 2 and 3. *P(I,J,K,L,M)* is the relative frequency of the *I,J,K,L,M* genotype where, *I*, *J*, *K*, *L* and *M* take values 1, 2, or 3 and *∑_I_∑_J_∑_K_∑_L_∑_M_ P(I,J,K,L,M) = 1*; there are 3^5^ = 243 possible genotypes. We assume that the fitness of a genotype is proportional to the sum of the values of the alleles to some power, *e (e≥1)*, with all five genes contributing equally to fitness. We also assume that the contribution of the number 2 allele at any locus is intermediate between that of the number 1 and number 3 alleles. Thus the fitness of a genotype I,J,K,L,M,
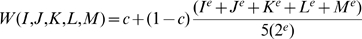
Where *(1−c)* is a measure of the extent to which these five loci contribute to fitness. For example with *c* = 0, and *e* = 1, the fitness of a genotype, 1,2,2,3,1 would be 9/10 = 0.90. The total range of fitness values would be 0.5 to 1.5, with 1,1,1,1,1 being the genotype of lowest fitness and 3,3,3,3,3 being the genotype of highest fitness. If *c* = 0.5 and *e* = 2, the range of fitness values would be 0.625 for the 1,1,1,1,1 genotype to 1.625 for the 3,3,3,3,3 genotype. With this fitness function, the 2,2,2,2,2 genotype would have a relative fitness of 1.0 independently of the value of *e*. The course of this simulation is diagrammed in Figure 1. In the following we describe the different steps in the simulation

#### Mutation

For individuals of all genotypes there is a probability μ per cell per generation of a mutation occurring in one of the five loci. For convenience we assume there is one generation per hour. To simulate this process, at each time interval, *Δt*, the probability that a mutant will be generated is PM = N*μ*Δt, where *N bacteria per ml* is total number of individuals in the population as well as the density. A pseudo random number, *r*, from a rectangular distribution *0≤r≤1* is generated. If *r≤PM*, a mutation occurs **(YES)**, if *r>PM* it does not **(NO)**. In these simulations, we use values of *N* and *Δt* such that at any given time *PM<1*. If a YES decision is made, we select the genotype that may be changed by mutation. For this and similar decision processes, a pseudo random number is generated and sequentially compared to the sum of the probabilities of the different outcomes, which in this case are frequencies of the different genotypes. The cumulative sum of the frequencies of the different genotypes are continually calculated, *P(1,1,1,1,1)+P(1,1,1,1,2)+P(1,1,1,1,3)* … and as soon as that sum exceeds the value of the random number, *r*, the process terminates, and the last genotype in the sequence is chosen to receive the mutation. Using a similar Monte Carlo decision process we then choose the locus for the mutation under the assumption that each locus has the same probability of being replaced, *y = 1/5*. For example, if *r = 0.4567*, the third locus, *J*, is subject to change by mutation and if *r = 0.9532*, the fifth locus, *M*, is subject to change by mutation. Following the choice of the genotype and locus for mutation, another Monte Carlo decision process is used to determine the allelic state of the mutation. Here we use three probabilities, *xm_1_*, *xm_2_ and xm_3_*, for the comparison, where *xm_1_+xm_2_+xm_3_*, = 1.0. In this simulation a mutation need not result in the change an allele. For example, if the 3^rd^ locus is chosen and bears a 2 allele, there is a probability xm_2_ that that allele remains in a 2 state. Upon choosing the genotype to be changed, the frequency of the mutated genotype is reduced by *1/N* and that of the mutant type is increased by *1/N*.

#### Recombination loop

As in [28] we assume recombination is a mass action process that occurs at a rate proportional to the product of the densities of the donor and recipient populations. We also assume that: (i) the five loci are sufficiently far apart that only a single gene is replaced in any HGR event, (ii) all loci have equal probabilities of being subject to allelic replacement by recombination, (iii) all individuals in the population are equally likely to serve as a donor and recipient. Thus, if the density of the population is *N*, the probability of a recombination event occurring during the finite time interval *Δt* is *PR* = χ*N^2^Δt where *χ ml/cell^2^/hour* is the rate parameter of recombination for those five loci [28]. When the value of *χ* is relatively low, *Δt* is set so that *0≤PR<1* and the decision regarding whether recombination occurs is random. If the random number *r≤PR*, recombination occurs **(YES)** and if *r>PR*, it does not occur **(NO)**. To reduce the number of random numbers generated, when *χ* is larger (usually 5×10^−14^ or greater) the recombination decision process is deterministic, and χ*N^2*^Δt recombination events occur during the interval *Δt*.

Independently of the value of *χ*, the choice of the recipient, donor, locus and allele for recombination is stochastic. For this we use the random number decision process similar to that described above for choosing the genotype subject to mutation. The final step in the recombination loop is to choose the locus in the recipient that is subject to replacement by that locus from the donor. The recombination loop terminates by increasing the frequency of the recombinant genotype by *1/N* and reducing that of the recipient genotype by *1/N*. The frequency of the donor genotype remains unchanged.

#### Selection loop

The mean fitness of all the genotypes is calculated as the sum of the product of the post mutation post recombination frequencies of each genotype and their relative fitness,
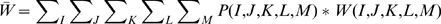
Where I,J,K,L, and M take values 1, 2 or 3.

The frequency of that genotype in the next time interval *t+Δt* is then calculated as
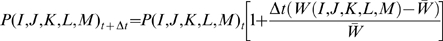



Once this is done for all genotypes, a new cycle of mutation, recombination and selection commences and the relative frequencies of the different genotypes in the population are adjusted accordingly. Unless otherwise indicated, iterations through this simulation continue for a defined number of generations and a defined number of runs.

#### Two competing populations

To explore the conditions under which recombination will be favored in the presence of a competing population with a different rate of recombination, we allow for a second population. The progress of both populations through the mutation, recombination, and selection process are identical to that described above for a single population. The two competing populations can, however, have different fitness functions, so that
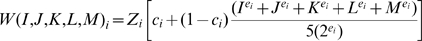



The parameters *Z_i_* (i = 1, 2) are measures of the intrinsic fitness of two populations *(0≤Z_i_≤1*) and the *e_i_*'s are the exponents. In these two population simulations we also allow for different rate parameters of recombination, *χ_1_* and *χ_2_* and different densities, *N_1_* and *N_2_*, but maintain a constant total density *N_1_+N_2_ = N_T_*. In these simulations both the recipients or donors can come from the same population or the donors can come from either population. In the former case, the rate of recombination is proportional to the square of the densities of the population in which recombination is occurring, *N_1_^2^* or *N_2_^2^*. In the latter case, the rate of recombination is proportional to the product of the density of the recipient population and the total density, N_1_*N_T_ and N_2_*N_T_. In situations where all cells in the population can serve as donors, the likelihood of a population serving as a donor is proportional to its frequency in the community.

The densities of these two populations and thus the relative frequencies of these genotypes can change by clone level selection. For this we assume the mean fitness of the two populations 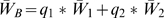


Where 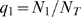 and 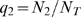 (since *N_1_+N_2_ = N_T_*, *q_1_+q_2_ = 1*).

For the next time interval, *t+Δt*,
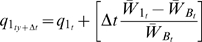
and
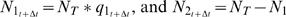



#### Starting conditions

The simulation runs to be considered were started in one of two ways: (i) with only a single genotype of intermediate fitness, *P(2,2,2,2,2)* = 1 or (ii)with a random selection of *nc* genotypes, (1<*nc*<3^5^) for each of the two populations. For the second starting condition (ii) the random number decision process was run *nc* times each time with picking an allele 1,2 or 3 at each of the five loci and assigning that genotype a random number *(0<r<1)*. If the same genotype is selected multiple times, its relative frequency would be proportional to the sum of the random numbers chosen for that genotype. In simulations initiated with the second starting condition the relative frequencies of the genotypes in the population are normalized by dividing by the sum of the frequencies. Unless otherwise noted, at the start of each of the runs made with two competing populations, both initially had the same *nc* clones although the clones chosen varied between runs.

#### Parameter values

The total population size was maintained at 10^8^ for simulations with single populations and at 2×10^8^ for the simulations with two competing populations. Although we use different rates of mutation μ = 10^−8^, 10^−7^ and 10^−6^, in all runs we assume that mutations to the lower fitness 1 allele occur at a rate greater than that for intermediate fitness allele 2, which in turn occur at a rate greater than that for the highest fitness 3 allele, i.e. *xm_1_* = 0.80, *xm_2_* = 0.15 and *xm_3_* = 0.05 independently of the existing state of the allele. The recombination rate parameters in these simulations are within range anticipated for *E. coli*, *H. influenzae*, *B. subtilis* and *S. pneumoniae* (see the Discussion).

**Figure 2:**
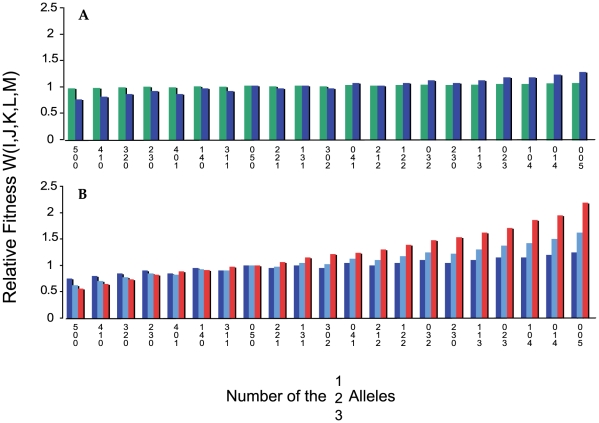
Relative fitness of the different genotypes *W(I,J,K,L,M)* as a function of the number of 1, 2 and 3 alleles as calculated from equations (1). (A) green: c = 0.9, e = 1.0, blue: c = 0.5, e = 1.0; (B) blue: *c* = 0.5, e = 1.0, light blue: c = 0.5, e = 2.0, red: c = 0.5, e = 3.0.

In Figure 2, we illustrate the relationship between the number of 1, 2 and 3 alleles and the fitness *W(I,J,K,L,M)* of the different genotypes for the different simulations employed. For example, the genotype (1,3,2,2,1) has 2 #1 alleles, 2 #2 alleles and 1 number #3 allele and in this figure would be designated as 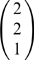. With the parameters, c = 0.5 and e = 2, the fitness of cells of this (1,3,2,2,1) genotype relative to the (2,2,2,2,2) genotype 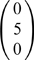 would be, from equation 1.
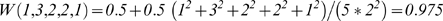



In Figure 2A, the exponent, *e = 1*, and the contribution of the five loci to fitness are either 0.1 or 0.5. In Figure 2B, the contribution of the five loci to fitness is 0.5, and the exponent e takes values1, 2, and 3. As this exponent increases the contribution of the higher index allele 3 to the fitness of a genotype increases, as does the magnitude of the fitness differential.

#### Simulations

The programs for this simulation study were written in quaint but fast FORTRAN 77. Copies of the FORTRAN code and/or executable files for Windows and Mac and instructions for their use are available from http://www.eclf.net.

## Results

We open our analysis with a consideration of the contribution of recombination to the rate of increase in the average fitness of single populations of bacteria. In these simulations, the five loci contribute equally to fitness and the three alleles at each locus, 1, 2 and 3 contribute additively. Mutation and recombination are random processes with all five loci equally likely to change in any given mutation or recombination event. In the case of mutation, the change in allelic state is independent of the genetic structure of the population. For recombination, the likelihood of a particular change in the allelic state of any of the five genes in recipient is proportional to the frequencies of those alleles in the population at large. Selection is a deterministic process with fitness being proportional to the frequency of high index alleles (see Figure 2). (For more details we encourage the reader to at least peruse the METHODS section, which we believe is written a way that would be amenable to those who prefer to hum equations than solve them.)

**Figure 3:**
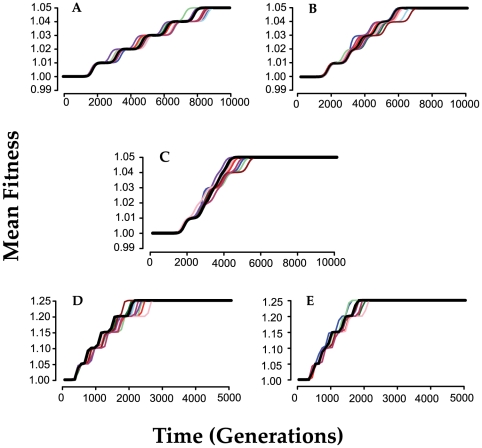
Change in mean fitness with different rates of recombination for ten independent runs initiated with a population monomorphic for the 2,2,2,2,2 genotype. Parameters, *N = 10^8^*, *μ = 10^−8^*, *xm1 = 0.80*, *xm2 = 0.15*, *xm3 = 0.05*, *c = 0.90* in (a), (b), and (c) and *c = 0.50* in (d) and (e) the exponent e = 1. (a) No recombination, (b) *χ* = 10^−15^, (c) *χ* = 10^−14^, (d) no recombination, (e) *χ* = 10^−15^. The thick dark line in these figures is the mean of the 10 runs.

**Figure 4:**
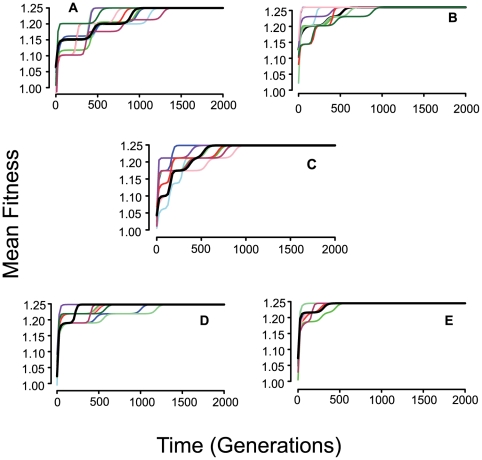
Increase in mean fitness for 10 independent runs with random start of nc = 10 or 50 clones. In all cases, *c = 0.5*, *e = 1*, *μ = 10^−8^*, *N = 10^8^*. (A) *c* = 0, *nc = 10* (B) *c = 10^−15^ nc = 10*, (C) *χ* = 10^−14^, nc = 10, (D) *χ = 0*, *nc = 50*, (e) *χ* = 10^−15^, *nc = 50*. The thick dark line is the mean fitness of all 10 runs.

The rate at which a population adapts to its environment, as measured by the increase in its mean fitness is directly proportional to the rate of recombination and the relative magnitude to which the five loci contribute fitness, as measured by the parameter 1−*c* (Figure 3). If there is more variation in the population at the start of a simulation, as there is when we start with 10 or 50 randomly selected lineages (Figure 4), the rate of evolution is faster than when the population is initially monomorphic as in Figure 3. This is of course anticipated from Fisher's fundamental theorem of natural selection [29] as well as simple logic.

**Figure 5:**
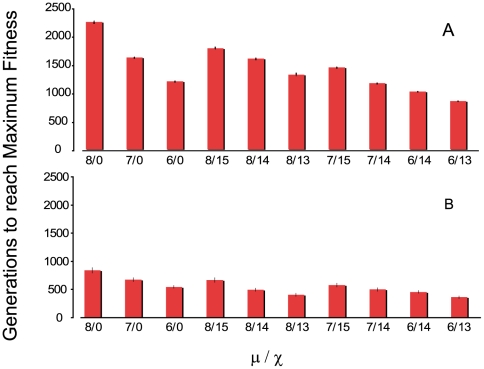
Time to reach the maximum mean fitness 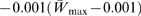 for different rates of mutation and recombination. For the mutation rates (μ) −8 is 10^−8^, and −7 is 10^−7^ per cell per hour. For the recombination rate constants (*χ*), 0 is no recombination, −15 is χ = 10^−15^, −14 is χ = 10^−14^. In all simulations *c = 0.5*, *e* = 1 and density and total population size is 10^8^ cells per ml. Means and standard errors of the time to reach maximum fitness for 50 runs with each set of parameters. (A) Runs initiated with a single intermediate fitness clone, 2,2,2,2,2. (B) Runs initiated with 10 randomly chosen clones.

To provide an overview of the relative contributions of mutation and recombination to the rate of evolution in this model, we did fifty simulations with different mutation and recombination rates. We started these runs with either a single genotype of intermediate fitness, 2,2,2,2,2 genotype, or 10 randomly chosen clones (Figures 5A and 5B, respectively). In these figures we plot the mean and standard error of the time required for the mean fitness of the population to reach 0.001 less the maximum fitness, 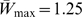. As anticipated from the results presented in Figures 3 and 4, the time to reach maximum fitness decreases with the rates of recombination and mutation. Saying this another way the rate of adaptive evolution increases with the rate of recombination and mutation. Although the mutation process is biased towards generating lower fitness alleles, with only five loci and the fitness and other parameters employed, the effects of generating less fit mutations on the average fitness are imperceptible. This is the case even with a mutation rate of 10^−5^, all of the variation in fitness determined by these five loci, *c = 0*, and populations initiated with the highest fitness genotype 3,3,3,3,3 (data not shown). Although lower fitness mutants are produced, they are purged by “purifying” selection and do not accumulate.

**Figure 6:**
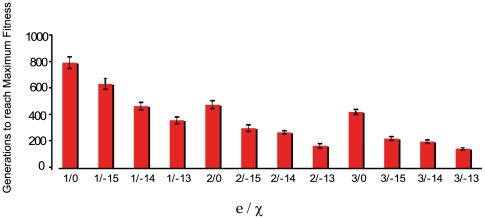
The contribution of intensity and form of the fitness function on the extent to which mutation and recombination augment rates of adaptive evolution; mean and standard error of the time before the population reaches its maximum value 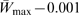 for different values of the exponent e, and recombination rate parameter (*χ*). For the latter −15 is χ = 10^−15^ and −14 is *χ* = 10^−14^. In all simulations, *μ = 10^−8^*, *N_T_ = 10^8^*, *c = 0.5*. Each simulation was initiated with 10 randomly chosen genotypes and 50 independent simulations were run with each set of parameters.

As noted in Figure 3, the extent to which recombination increases the rate of evolution is proportional to the intensity of selection at the loci subject to recombination, the selection differential. To explore this relationship a bit more and begin to consider the contribution of the form of the fitness function, we have performed simulations with *c = 0.5* and exponents *e = 1*, *e = 2* and *e = 3* (see Figure 2B). As *e* increases, the contribution of the higher number alleles becomes proportionally greater and the time to reach maximum fitness is reduced. The results of these “experiments” are presented in Figure 6.

To provide a more detailed view of the contribution of the initial variability to the effects of recombination on the rate of evolution, we made 50 runs with each set of parameters. Each run would terminate when the mean fitness was nearly its maximum 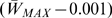 or 5000 generations passed. The results of these simulation experiments are presented in Table 1. To better evaluate the relative contributions of the initial variability and the rate of recombination, we performed a two-way analysis of variance (ANOVA) on the first three rows (2 df) and four columns (3 df). Increasing the amount genetic variability in the population at the start of each run and the rate of recombination significantly increases the rate of evolution, (*p<10^−16^*). There is also a significant interaction *p<0.005* for the combined effects of initial variability and rate of recombination.

**Table 1:** The contribution of the initial number of clones and the rate of recombination to the time before the population reaches its maximum fitness 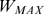 (mean±standard error to the nearest generation).

Initial Clones	χ = 0	χ = 10^−15^	χ = 10^−14^	χ = 10^−13^
1*	2248±24	1785±17	1603±22	1383±31
10	935±49	668±44	509±29	450±26
50	626±41	395±27	322±22	278±15

Standard parameters, *c* = 0.5, *μ* = 10^−8^, *xm1* = 0.80, *xm2* = 0.15, *xm3* = 0.05.

*All simulations were initiated with only the 2,2,2,2,2 genotype.

### Competition between Populations With Different Rates of Recombination

To explore the conditions under which the capacity for homologous gene recombination will provide an advantage to a population, we consider mixtures of two genetically distinct populations, one of which does not recombine (in which variation is only generated by mutation), or recombines at a lower rate than the other. For each population, mutation, recombination and selection occur as described for the single population simulations. Although the total density of the two-population community remains constant, the densities and relative frequencies the two competitors change at a rate that depends on their respective mean fitness. Unless otherwise stated, recombination only occurs within a population. In these simulations, the rate parameter of recombination of the #1 population exceeds that of the #2 population, *χ_1_>χ_2_* and in most cases *χ_2_* is 0.

**Figure 7:**
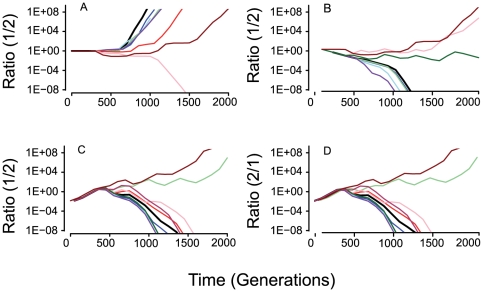
Competition between populations with different rates of recombination. In (A), (B) and (C) we plot the changes in the ratio of the higher rate recombining population #1, relative to the lower rate recombining population, #2, 1∶2. In Figure (D) we plot change in the ratio of non-recombining #2 to recombining populations, #1, 2∶1. The results of 10 independent runs for each initiated with a monomorphic, 2,2,2,2,2 population. In the runs depicted the total population size *N_T_ = 2×10^8^*, *μ = 10^−8^*, *χ_1_ = 5×10^−15^*, *χ_2_ = 0*, *c = 0.50*, *e = 1.0*. (A) Initially equal densities of #1 and #2 and no fitness cost associated with recombination. (B) Initial equal frequencies of #1 and #2 and a 1% fitness cost for the recombining population #1. (C) The recombining population is initially rare and there is no fitness cost associated with recombination. (D) The non-recombining population is initially rare and there is a 2% fitness cost associated with recombination.

In Figure 7, we follow the changes in the ratio of the two populations for different situations with the #1 and #2 populations initially monomorphic for the intermediate fitness genotype, *2,2,2,2,2*. If there is no cost to recombination and initially the #1 and #2 populations are equally frequent, the recombining #1 population has an advantage over the one that is not recombining, #2 (Figure 7A), i.e. in 9/10 runs the recombining populations prevailed. When there is 1% fitness cost associated with recombination and initially equal frequencies of the #1s and #2 populations, in the majority of runs the non-recombining population has an advantage (Figure 7B).

Although in the absence of an intrinsic fitness cost, HGR provides a clear advantage when the recombining population is common, this is not necessarily the case when the recombining population is initially rare (Figure 7C). On the other hand, the capacity for HGR can prevent the establishment of an initially rare, higher fitness, non-recombining population (Figure 7D).

**Figure 8:**
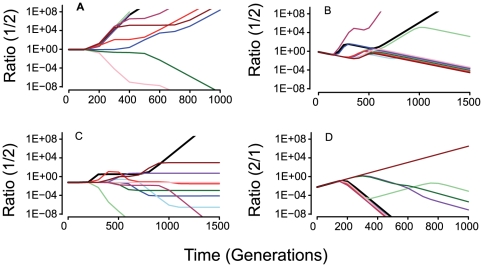
Competition between populations with different rates of recombination. In (A), (B) and (C) we plot the changes in the ratio of the higher rate recombining population #1, relative to the lower rate recombining population, #2, 1∶2. Runs initiated with 10 randomly chosen clones with identical starting populations for the 1 and 2 populations. In all runs the runs depicted the total population size *N_T_ = 2×10^8^*, μ = 10^−8^, *χ_1_ = 5×10^15^*, *χ*_2_ = 0, c = 0.50, e = 1.0. (A) Initially equal densities of 1 and 2 and no fitness cost associated with recombination. (B) Initial equal frequencies of #1 and #2M and a 1% fitness cost for the recombining population 1. (C) The recombining population is initially rare and there is no fitness cost associated with recombination. (D) Ratio of non-recombining to recombining 2∶1, the non-recombining population is initially rare and there is a 2% fitness cost for the recombining population.

In the simulations described above (Figure 7) the population are initially monomorphic and recombination does not come into play until sufficient variation builds up through recurrent mutation (see Figure 3). Qualitatively, the results obtained with runs stated with 10 randomly selected clones are similar to those initiated with no variability (compare Figures 7 and 8), but there are quantitative differences. The most conspicuous of the quantitative differences between the results presented in Figure 7 and Figure 8 is that when the populations are initially variable, the outcome of competition is more likely to end in a stalemate than the loss of the #1 or #2 population. This was particularly true for the runs initiated with a rare recombining population (Figure 8C). The reason for this stalemate is that the two populations both reach the maximum fitness before the run terminates and selection ceases.

To provide a broader and more quantitative perspective of the effects of recombination on the outcome of competition, including invasion and prevention of invasion, we made 50 independent runs with different rates of recombination, different initial frequencies of the #1 and #2 populations and different fitness costs. As controls for these “experiments” we used 100 simulations with the two populations having the same recombination rates. These simulations were run until the density of one population fell below 10^5^ (the total density remained constant at 2×10^8^) or 2000 generations passed. The results of these experimental and control simulations are presented in Table 2. In these simulations a population “won” the competition when the density of the other population fell below 10^5^ or when it had the highest relative frequency after 2000 generations passed.

**Table 2:** Competition between a recombining (#1) and non-recombining population (#2): effects initial variability, recombination rate, and fitness costs of recombination.

Runs initiated with a monomorphic 2,2,2,2,2 population Freq of #1/*χ*/s	Runs where # 1 Wins (TF±SE)	Runs where # 2 Wins (TF±SE)	Runs with #1 Winning at T = 2000	Runs with #2 Winning at T = 2000
0.5/5×10^−15^/0.01	**50** * (1180±60)	**0**	**0**	**0**
0.05/5×10^−15^/0.01	**9** * (1151±9 )++	**41** (726±47)	**0**	**0**
0.005/5×10^−15^/0.01	**0**	**50** (424±18)	**0**	**0**
0.5/5×10^−15^/0.01	**2** (1351)	**47** (747±33)	**0**	**1**
0.5/5×10^−15^/0.02	0	50 (391±82)	0	0
0.5/5×10^−14^/0.02	0	50 (389±5)	0	0
0.5/5×10^−13^/0.02	0	50 (378±2)	0	0
0.95/5×10^−15^/0.02	**1**	**48** (720±35)	**0**	**1**
0.95/10^−8^/5×10^−14^/0.02	**5** (960±65)++	**43** (732±47)	**1**	**1**
0.95/10^−8^/5×10^−13^/0.02	**23** * (1023±47)++	**25** (739±34)	**2**	**0**
0.5/5×10^−15^/0.01 Cont.	**0**	**100** (642±8)	**0**	**0**
0.5/5×10^−15^/0.02 Cont.	**0**	**100** (398±4)	**0**	**0**
0.05/5×10^−15^/0.00 Cont.	**0**	**100** (479±12)	**0**	**0**
0.005/5×10^−15^/0.00 Cont.	**0**	**100** (432±7)	**0**	**0**

Runs initiated with 10 random clones Freq #1/*χ*/s	Runs where # 1 Wins (TF±SE)	Runs where # 2 Wins (TF±SE)	Runs with #1 Winning at T = 2000	Runs with #1 Winning at T = 2000
0.5/5×10^−15^/0.01	**37** * (453±30)	**3**	**9**	**1**
0.05/5×10^−15^/0.01	**8** * (675±101)	**28** (521±44)	**9**	**5**
0.005/5×10^−15^/0.01	**0**	**50** (424±18)	**0**	**0**
0.5/5×10^−15^/0.01	**24** * (533±50)++	**21** (1421±80)	**0**	**5**
0.5/5×10^−15^/0.02	**11** * (608±108)	**39** (739±38)	**0**	**0**
0.5/5×10^−14^/0.02	**21** * (523±62)++	**28** (1070±81)	**0**	**1**
0.5/5×10^−13^/0.02	**28** * (425±36)++	**22** (942±75)	**0**	**0**
0.95/5×10^−15^/0.02	**31** * (541±57)++	**19** (1110±101)	**0**	**0**
0.95/5×10^−14^/0.02	**41** * (390±29)++	**9** (1024±123)	**0**	**0**
0.95/5×10^−13^/0.02	**40** * (313±22)++	**10** (1073±86)	**0**	**0**
0.5/5×10^−15^/0.01 Cont.	**0**	**100** (671±19)	**0**	**0**
0.5/5×10^−15^/0.02 Cont.	**0**	**100** (365±3)	**0**	**0**
0.05/5×10^−15^/0.00 Cont.	**0**	**82** (415±18)	**0**	**18**
0.005/5×10^−15^/0.00 Cont.	**0**	**100** (279±9)	**0**	**0**

Number of times each population Wins (the density of the competing population falls below 10^5^) or is Winning (is the dominant population at the 2000^th^ generation) in 50 or 100 independent runs. TF is the mean number of generations before the density of the losing population falls below 10^5^ ±SE: standard error.

In all these simulations, ***μ = 10^−8^***, ***c = 0.5***, ***e = 1***, and **N_T_ = 2×10^8^**. The initial frequencies and selection coefficients, *s*, are for the **#1** population. The simulations labeled **Cont.** are controls in which both the #1 and #2 populations recombine with a rate constant X = 10^−15^. In the experimental runs, there is no recombination in the # 1 population.

*significant with p<0.002 (X^2^ contingency table comparing number of wins of #1 and #2 in treatments vs. control).

**++:** significant with p<0.005 (Student's t-test comparing TF between Population 1 and Population 2 within treatment).

For the simulations initiated with a monomorphic, all genotype *2,2,2,2,2* populations, in the absence of a cost and initially equal frequencies, the recombining population “won” in all 50 simulations. When the initial frequency of the recombining population was 0.05, the recombining population won about 20% of the time. When the initial frequency of the #1 was 0.005, the non-recombining #2 won in all 50 simulations. In the parallel no-fitness-cost runs with the initially variable population and initially equal densities in the majority (but not all) of the runs the #1 populations “won” or was “winning” when the runs terminated at 2000 generations. These runs illustrate how the advantages of recombination are reduced when the initial frequency of the recombining population is lower than that of the non-recombining population. The largest quantitative difference between the populations with and without initial variability is in the time required for this outcome (winning) to obtain, which, as suggested by the single population runs, is longer in the initially monomorphic populations. That recombination was responsible for the winning # 1 population in these no-fitness-cost runs with the recombining population initially rare, can be seen from the controls where there was no recombination in the # 1 population.

With a 1% or 2% fitness cost associated with recombination, even when the recombining and non-recombining populations are initially equally frequent, the non-recombining population almost invariably prevails when the competitors are initially monomorphic. A very different situation obtains when at the start of the competition there is genetic variability (10 randomly selected runs). Under these conditions even with the lowest rate of recombination examined, *χ* = 5×10^−15^, a substantial fraction of the recombining populations wins even in the face of a 2% cost in intrinsic fitness. Moreover, the time before the recombining population wins is significantly shorter than that in the runs where the non-recombining, #2, population wins. This effect of initial variability also obtains in situations where the non-recombining population is initially common. With an initially variable population, recombination provides a substantial advantage in competition with a rare but intrinsically fitter population. This is less so when the population is initially monomorphic. But even then with a sufficiently high rate of recombination the #1 population can prevail in competition with a high fitness non-recombining population.

To obtain more information about the contribution of the intensity and form of the fitness function to the conditions under which within-host selection would favor recombination, we performed simulated competition experiments with different values of the exponent ***e***. As noted in Figure 5A, the intensity of selection due to these five loci and the contribution of the highest fitness 3 allele to that increase is directly proportional to ***e***. The results of these simulations are presented in Table 3.

**Table 3:** Competition between a recombining (#1) and non-recombining population (#2): effects of initial variability, recombination rate, fitness costs and the relative contributions of the higher fitness alleles (e) on the outcome of competition.

Runs initiated with a monomorphic 2,2,2,2,2 population Freq of #1/*χ*/s/e	Runs where # 1 Wins (TF±SE)	Runs where # 2 Wins (TF±SE)	Runs with #1 Winning at T = 2000	Runs with #2 Winning at T = 2000
0.5/5×10^−15^/0.02/3	18* (241±22)	32 (197±12)	0	0
0.5/5×10^−14^/0.02/3	23* (253±18)	27 (255±53)	0	0
0.5/0/0.02/3	5 (583±95)++	45 (352±28)	0	0
0.5/5×10^−15^/0.02/2	12* (283±41)	38 (230±18)	0	0
0.5/5×10^−15^/0.02/1	0	50 (397±6)	0	0
0.05/5×10^−15^/0.00/3	3 (1151±9)++	47 (198±14)	0	0
0.005/5×10^−15^/0.00/3	0	47 (103±7)	0	3
0.95/5×10^−15^/0.02/3	41* (183±14)	9 (391±119)	0	0

Runs initiated with 10 random clones Freq #1/*χ*/s/e	Runs where # 1 Wins (TF±SE)	Runs where # 2 Wins (TF±SE)	Runs with #1 Winning at T = 2000	Runs with #1 Winning at T = 2000
0.5/5×10^−15^/0.02/3	36* (222±22)++	14 (725±61)	0	0
0.5/5×10^−14^/0.02/3	42* (162±7)++	8 (761±70)	0	0
0.5/0/0.02/3	5 (376±48)	45 (299±30)	0	0
0.5/5×10^−15^/0.02/2	28* (277±19)++	22 (691±68)	0	0
0.5/5×10^−15^/0.02/1	7* (472±95)++	45 (851±53)	0	0
0.05/5×10^−15^/0.00/3	11* (293±24)	30 (236±20)	4	5
0.005/5×10^−15^/0.00/3	0	43 (140±5)	0	7
0.95/5×10^−15^/0.02/3	46* (149±5)++	4 (596±28)	0	0

Number of times each population Wins (the density of the competing population falls below 10^5^) or is Winning (is the dominant population at the 2000^th^ generation) in 50 independent runs. TF is the mean number of generations before the density of the losing population falls below 10^5^ ±SE: standard error.

In all these simulations, *μ = 10^−8^*, *c = 0.5*, and *N*_T_ = 2×10^8^ and for the experimental populations the recombination rates, *χ* and selection coefficient, *s*, are for the #1 population. The parameter ***e*** determines the magnitude of the contribution of each allele (see Figure 2).

*significant with p<0.002 (*χ* ^2^ contingency table comparing number of wins of the # 1 and #2 population in recombining vs. control non-recombining).

**++:** significant with p<0.005 (Student's t-test comparing TF between Population #1 and Population # 2 for a given set of runs).

The effects of having a greater range of fitness values and a greater contribution of the highest fitness allele, 3, can be seen by comparing the simulation runs in Table 3 with the parallel runs in Table 2. Most importantly, with a greater fitness range associated with these 5 loci and proportionally greater contribution of the #3 allele, when the competing populations are of roughly equal frequency, the increase in the rate of adaptation due to recombination is more likely to overcome the fitness burden associated with recombination it would with a more modest fitness range. This is true not only for the simulations initiated with genetically variable populations but also for those initiated with monomorphic, 2,2,2,2,2 populations (compare the outcomes of the ***e*** = 1 runs in Table 2 with the corresponding ***e***>1 simulations in Table 3).

On the other hand, even in the absence of a fitness cost associated with recombination and a greater contribution of these 5 loci and the number 3 allele to fitness, this capacity for gene shuffling does not enable the recombining population to invade when its frequency is low, 0.005. Although a greater contribution of the number 3 allele to fitness and a greater fitness differential associated with these five loci augments the likelihood of the recombining population winning over an initially rare but higher fitness, non-recombining competitor.

### Competition between Genetically Different Populations

In the preceding, the recombining and non-recombining populations were at the start of each run genetically identical, either monomorphic for the same genotype, 2,2,2,2,2 or had the same set of genetically variable clones. To begin to explore the more realistic situation where the competing populations with and without sexual proclivity are initially different genetically, we performed simulations with 10 initially different random collections of genotypes for #1 and #2 populations. The results of these simulations are presented in Table 4.

**Table 4:** Competition between a recombining (#1) and non-recombining population (#2): effects of the recombination rate, fitness costs and the initial frequency of the recombining population on the outcome of competition.

Initial Freq #1/s/*χ*	Runs where # 1 Wins (TF±SE)	Runs where # 2 Wins (TF±SE)	Runs with #1 Winning at T = 2000	Runs with #2 Winning at T = 2000
0.5/0.00/0.00	47 (102±13)	51 (141±19)	1	1
0.5/0.00/5×10^−15^	73* (110±8)	24 (75±9)	3	0
0.5/0.00/5×10^−14^	74* (102±8)	24 (78±13)	2	0
0.5/0.00/5×10^−13^	66* (100±9)	29 (71±11)	4	1
0.05/0.00/0.00	38 (101±14)	60 (109±13)	1	1
0.05/0.00/5×10^−14^	56* (134±14)++	40 (57±9)	4	2
0.5/0.02/0.00	44 (103±17)	56 (130±21)	0	0
0.5/0.02/5×10^−14^	54 (134±4)++	40 (57±9)	0	0
0.5/0.10/0.00	18 (175±39)++	82 (94±11)	0	0
0.5/0.10/5×10^−14^	30 (141±24)	70 (100±11)	0	0

Number of times each population Wins (the density of the competing population falls below 10^5^) or is Winning (is the dominant population at the 2000^th^ generation) in 100 runs. TF is the mean number of generations before the density of the losing population falls below 10^5^ ±SE. In these simulations the at the start of each run the #1 and #2 populations have genetically different populations.

In all these simulations, **μ = 10^−8^**, **c = 0.5**, **e = 1.0**, and **N_T_ = 2×10^8^** and for the experimental populations the recombination rate parameter χ and fitness costs, *s*, are for the recombining #1 population.

*: significant differences with p<0.002 for a Fisher exact test for the number of Wins of population #1 and #2 relative to the corresponding runs without recombination.

**++:** significant, p<0.005 (Student's t-test comparing the TF between Population #1 and Population #2 in a given sent of runs).

In the absence of selection against recombination and initially equal frequencies of the #1 and #2 populations, as measured by the relative numbers of winners and losers the recombining population has an advantage over the non-recombining despite the initial genetic differences between these populations. This can be seen, by comparing the simulations for the recombining “experimental” populations (rows, 2, 3 and 4) and the non-recombining control (row 1). Notably, the rate of recombination seems to have no effect on the frequency of winning. Under these conditions, the initial fitness of the competing populations plays a more prominent role in determining the outcome of competition than the increase in fitness occurring during course of competition. By this same winning and losing criteria, in the absence of an intrinsic fitness cost, recombination increases the likelihood of the #1 population ascending to dominance when it is initially relatively rare (0.05). On the other hand, with initial genetic differences in the recombining and non-recombining populations and a cost associated with capacity for recombination, HGR does not provide a statistically significant advantage for the #1 population. Moreover, with initial differences in the genetic composition of the recombining and non-recombining populations, the time to winning by the non-recombining population is less than that of the recombining.

### Both Populations Can Serve as Donors

In all of the preceding runs, we assumed that recombination only occurs within a population. It may well be that both populations can contribute as donors even when they both cannot serve as recipients, e.g. when recombination is through the uptake of exogenous DNA, transformation. To explore this situation, we used a version of the simulation where the donors for recombination can be chosen from both populations, with the choice dependent solely on their relative frequencies.

In Table 5 we compare the outcomes of simulations where only members of the recombining population #1 serve as donors as well as recipients with corresponding situation where members of both populations can serve as donors. In the simulation results presented in this table, the recombining population has a 2% intrinsic fitness cost. In the runs where the initial frequency of the recombining populations was 0.05, the acquisition of genes from the non-recombining population increased the likelihood of invasion. Although with a 1∶1 ratio there was a significantly higher frequency of recombining populations winning when both populations served as donors in the initially monomorphic runs, this was not the case for the simulations initiated with 10 randomly selected clones. On the other hand, when the initial frequency of the recombining population was 5% for both the initially monomorphic and polymorphic populations, the recombining population was more likely to win when both populations served as the source of genes for recombination. When both populations served as donors and both were initially polymorphic, winning by the recombining population took less time then it did for the winning non-recombining populations. It should be noted, however, that for any value of *χ* when both populations serve as donors because of the greater density of the population,the frequency of recombination was greater than when only one competitor served as the donor.

**Table 5:** Competition between a recombining (#1) and non-recombining population (#2): effects of the initial frequency of #1 and the both populations as donors on the outcome of competition. 50 independent runs with each set of parameters.

Relative frequency of #1 Mono or Polymorphic	Donor Populations	#1 wins (TF±SE)	# 2 wins (TF±SE)
0.05: Mono	1 only	2 (472±24)	48 (123±7)
	1 and 2	15* (312±21)++	35 (122±9)
0.05 – Poly 10	1 only	0	50 (354±39)
	1 and 2	11* (232±30)++	39 (603±34)
0.50 – Mono	1 only	32 (333±20)	18 (457±86)
	1 and 2	49* (222±5)	1 (227±10)
0.50 – Poly 10	1 only	29 (205±7)++	21 (670±38)
	1 and 2	34 (184±100)++	16 (726±34)

Number of times each population Wins (the density of the competing population falls below 10^5^) or is Winning (is the dominant population at the 2000^th^ generation). TF is the mean number of generations before the density of the losing population falls below 10^5^ ±SE: standard error.

In all runs there was a 0.02 fitness cost for the #1 population – *χ*_1_ = 5×10^−14^, μ = 10^−7^, N_T_ = 2×10^8^, c = 0.5, e = 3. In all runs both populations were initially only the 2,2,2,2,2 genotype; Poly 10 - both populations were initiated with the same10 randomly selected clones.

*p<0.002 (*χ* ^2^ contingency with Yates Correction).

**++:** significant with p<0.005 (t-student test comparing TF between Population 1 and Population 2 within treatment).

### Differences in Recombination Rates

The recombining and non-recombining populations considered in the preceding two population simulations are the extremes. It may well be that both competing populations are capable of recombination but do so at different rates. Based on the single population and the preceding mixed population results we would anticipate that if this were the case and all else were equal, the population with the higher rate of recombination would prevail. This is indeed confirmed by our simulation experiments. For example, for 100 simulations with initially monomorphic, intermediate fitness 2,2,2,2,2 populations in equal frequency, (*μ = 10^−7^,c = 0.5*, *e = 3*) and recombination rate parameters *χ_1_ = 5×10^−13^* and *χ_2_ = 5×10^−15^*, in 95 of the runs the #1 population won, or was winning at 2000 generation in the remaining 5 runs. On the other hand, with these starting conditions, when the initial densities of the #1 and #2 populations were respectively 10^7^ vs. 1.9×10^8^, the population with the lower recombination rate won in 99 out of 100 runs and the population with the higher rate of recombination won in only one run. The situation is different when both populations can serve as donors as well as recipients. In this 10^7^ vs. 1.9×10^8^ contest between the populations with high (#1) and low (#2) rates of recombination, the score for 100 runs were #1 won 45 times, #2 won 41 times, and the numbers of # 1s and #2s winning at the 2000 generation termination were, respectively 11 and 3.

## Discussion

We interpret the results of this computer simulation study as support for the proposition that that there are realistic conditions where homologous gene recombination (HGR) will increase the rate at which bacterial populations adapt to their environment. These results are also consistent with the hypotheses that by increasing rates of adaptive evolution, HGR can provide a population a selective advantage when competing with otherwise identical or even somewhat more fit populations that are unable to shuffle homologous genes or do so at lower rates. Our mixed population simulations, however, also illustrate a major caveat to the hypothesis that homologous gene recombination in bacteria evolved in response to selection for increasing rates of adaptive evolution. Even in the absence of a fitness cost, the recombining population will only have an advantage over a non-recombining population when the recombining population is relatively common; HGR will not be favored when it is rare.

### Homologous Gene Recombination and Rates of Adaptive Evolution

The validity and generality of these predictions are, of course, empirical questions. They are however, questions that can be addressed experimentally. And, as noted in our Introduction, there have been at least four experimental studies testing the hypothesis that recombination increases the rate at which bacterial populations adapt to culture conditions. The results of two of these experiments are consistent with this hypothesis, Cooper's study with F-plasmid-mediated recombination in *E. coli B* [24] and Baltrus and colleagues study of transformation-mediated recombination in *Helicobacter pylori* [25]. The results of the other two reports, Souza and colleague's study of Hfr-mediated recombination in *E. coli* [26] and Bacher and colleagues study of transformation-mediated recombination in *Acinetobacter baylyi* [27] are interpreted to be inconsistent.

How well do the results of this simulation study account for the outcomes of these recombination – rates of adaptive evolution experiments? We believe that at least at a qualitative level, the results of the three of these studies for which this model is a reasonable analog [24],[25],[27] are consistent with the predictions of these simulations. The format of the experiments by Souza and colleagues [26] were different from that of this model and therefore we do not believe these simulations are appropriate for interpreting their results. In their experiments, two genetically different *E. coli* strains were used; a Hfr strain of *E. coli* K-12 and a F- strain of *E. coli* B. Although the Hfr strain donated genes to the *E. coli* B, under the conditions of their experiments this donor did not replicate and it was not present throughout the course of the experiment as assumed in our model.

Although the details of the [24],[25],[27] experiments were different from those specified by this simple model, their basic structure was similar to that of the single population simulations initiated with monoclonal (2,2,2,2,2) populations. In these experiments, which were initiated with single clones of either recombining (Rec+ or Com+) or non-recombining (Rec− or Com−) populations, the bacteria were growing in liquid media and reached densities of 5×10^7^ per ml or greater. Although the rate constants of recombination *χ* were not estimated in these experimental studies, it was clear that recombination was occurring at a substantial rate. The frequency of gene replacement by recombination in the Rec+ *E. coli B* and Com+ *H. pylori* experiments exceed that expected by mutation, and in the Cooper study the rate of gene replacement by recombination greater is greater than that of the elevated rate of mutation of a *mutS* strain. For recombination mediated by HFR, F', F+ plasmid in *E. coli*, *χ*, it seems reasonable to conclude that in the Cooper experiments *c*>10^−13^ (Cornejo and Levin, In Preparation- but available, see www.eclf.net ). We would also expect *χ>10^−13^* for the *H. pylori* experiments and possibly in the *Acinetobacter baylyi* study as well. This is certainly the case for the only two experimentally obtained estimates *χ* we know of for transforming bacteria, *H. influenzae* [30] and *B. subtilis* [31], both of which are on the order of *χ*∼10^−12^.

With population densities, mutation and recombination rate constants in the ranges of these experiments, our simulations show that recombining populations evolved more rapidly than those that did not have this capacity for shuffling homologous genes. For any given mutation and recombination rate parameters, the rate and magnitude of increase in mean fitness depended on the fitness function. Cooper's observation that recombination increased the rate of adaptation to culture conditions with a higher mutation rate ∼3 times greater than it did with a lower rate [24] is also consistent with the predictions of this model; mutation and recombination act synergistically to increase rates of adaptive evolution. Although Bacher and colleagues [27] interpret the results of their experiments with *A. baylyi* to be inconsistent with the hypothesis that HGR increases rates of adaptive evolution, that is not the case for all the results they report. In their higher density experiments not only does the fitness of the population increase to a greater extent than in their low density experiments, but this increase in fitness was considerably as well as significantly greater (p = 0.00012 for a two tailed t-test) for the transformation competent population than the non-competent controls.

### Does Homologous Gene Recombination Increase Rates of Adaptive Evolution in Natural Populations?

While we are unaware of direct experimental evidence for an affirmative answer to this question from natural population studies, based on the predictions of the model we would anticipate a positive answer. Retrospective, multi-locus sequence studies suggest that the rates of gene of replacements by homologous recombination in species like *Streptococcus pneumoniae* exceed that by mutation by a factor of 10 or so [32],[33],[34], and are even greater for some species, like *H. pylori* [35],[36].

To put these retrospective estimates of recombination rates into the context of our model and its parameters, consider the following intuitive argument. Assume a 1-hour generation time, a habitat of 1 ml, a population of 10^8^ bacteria and a mutation rate of 10^−8^ per cell per generation. In the course of an hour in that population, for any given locus, an average of 1 mutant would be produced. If gene replacements by recombination occur at 10 times that rate, there would be 10 recombinants at that locus for a value of *χ* = 10/(10^8^×10^8^) = 10^−15^. As noted in our simulations, even at this low rate and an initially monoclonal population, recombination can increase the rate of adaptive evolution over that which would be anticipated by mutation alone. Moreover, natural populations of many bacteria are likely to be composed of multiple lineages and would be genetically variable at many loci. In accord with our simulations the pace at which recombination increases the rate of adaptive evolution would on average increase with the extent of genetic variability of the population, see Figure 5.

### Accelerating Adaptive Evolution as a Selective Force for the Maintenance and Evolution of HGR

Processes, like homologous gene recombination, that increase rates of adaptive evolution would be to the advantage of a population and augment its prognosis for surviving the vicissitudes of an ever-changing environment. This is, of course, the most common explanation for ubiquity of HGR among extant species of eukaryotes. Indeed, the presumed lack of recombination, sex to be more provocative, in ancient groups of seemingly successful organisms like the bdelloid rotifers make them intriguing objects for study [37]. Whether accelerating rates of adaptive evolution is the selective force responsible for the evolution and maintenance of recombination in eukaryotes is a subject of some controversy [20],[38], a subject that we are pleased to say is beyond the scope of this report. The population and evolutionary dynamics of recombination in bacteria are fundamentally different from that of sexually reproducing eukaryotes. In the bacteria, recombination depends on density and is not a part of the reproductive process. If they wish to procreate, sexually reproducing eukaryotes have no choice but to find mates and generate recombinant progeny, independently of density of their populations.

Here, we postulate that once the mechanisms for HGR are established in a bacterial population, the advantage accrued by a more rapid rate of adaptation to environmental conditions can promote their maintenance, even if they engender a modest cost in fitness. The necessary condition for this to obtain is that the adaptive process is continuous. This may be the case when a population enters a new environment and/or is confronted by either physical or biological factors that reduce the rates of survival or reproduction (the fitness) of its members. As long as the population is continually confronted with situations where selection favors new genotypes, as was postulated for evolution of mutator genes [39], recombination could continue to be favored and be maintained. This would not be the case if recombination engenders a fitness cost and the population is confronted with extensive periods of adaptive stasis. Under these conditions, the frequency of the recombining population will continue to decline. And, because of the frequency- and density- dependent nature of selection for recombination, the recombining population may not be able to recover. In this interpretation, the maintaining mechanisms of horizontal gene transfer by HGR by increasing rates of adaptive evolution is not an equilibrium outcome; on “equilibrium day” [40], recombination will be lost. Moreover, because HGR accelerating rates would not provide an advantage to a recombining population when it is initially rare, it is even less likely to have been a selective force for the original evolution of mechanisms for HGT than it is for maintaining those mechanisms once they evolved.

Models can be used to generate hypotheses and, in a quantitative way, evaluate their plausibility. They cannot be used to test those hypotheses! We are unaware of published empirical studies testing the hypotheses that selection for HGR is frequency- and density- dependent. These are, however, hypotheses that can be tested with experiments similar to the single clone studies testing the hypothesis that HGR increases rates of adaptive evolution [24],[25],[27]. The idea would be to follow the changes in frequency of Com+ or Rec+ in competition with Com− or Rec− clones with different initial frequencies of these competitors and in populations of different densities. We postulate that under conditions where they accelerate rates of adaptive evolution in single clone culture and adjusting for intrinsic fitness differences: (1) when introduced at roughly equal frequencies, the recombining population will have an advantage over a non-recombining competitor and, (2) the recombining population will not have that advantage when it is initially rare (in our simulations much less than 1%.). We also postulate that because of a lower rate of production of mutants as well as the lower frequency of recombination (which would be proportional to the square of the density of the recombining population); (3) the rate of adaptive evolution would be less in recombining populations of low density than otherwise identical populations of higher density and, (4) the minimum frequency for a recombining population to have a selective advantage in competition with one that cannot recombine would be inversely proportional to the total density of the recombining population.

Using the long-term evolved strains of *E. coli* B developed by Richard Lenski and colleagues [41],[42],[43] it should be possible to experimentally test the hypothesis that HGR will only be favored when there is relatively intense selection for adaptation to culture conditions. Although those experimental *E. coli* B populations continued to evolve in different ways as time proceeded the largest increase in mean fitness relative to the ancestral occurred within the first 5,000 or so generations. We postulate that if in an experiment similar to that in [24] the F'lac constructs were made with *E. coli* B taken from later generations, say >20,000, the recombining population will not evolve more rapidly than one that is not recombining.

### HGR and the Maintenance and Evolution of HGT in Bacteria

Two of the three major mechanisms responsible for HGT and HGR in bacteria, conjugation and transduction, are not properties of the bacteria but rather that of their parasites, primarily conjugative plasmids and bacteriophage. One needn't postulate that these processes evolved and are maintained by selection favoring bacteria with the capacity for HGT. The most parsimonious hypothesis for recombination mediated by plasmids and phage is as a coincidental byproduct of the infectious transfer of these elements and the host's recombination repair system [44],[45]. This would also be the case for recombination resulting from cell fusion [11] or transformation mediated by natural electroporulation or cold shocks. In this interpretation, accelerating the rate of adaptive evolution by HGR mediated by these processes are a lagniappe rather than a product of adaptive evolution. To be sure we can make up and probably construct mathematical models illustrating ways by which bacteria evolve mechanisms to be more receptive to plasmids and phage carrying genes on their behalf, but we see no need to stretch our imaginations in that direction.

The third main mechanism for HGT and HGR in bacteria, the uptake and incorporation of exogenous DNA, i.e. competence and transformation, are intrinsic properties of bacteria rather that of their parasites. We postulate that under some conditions HGR accelerating rates of adaptive evolution will promote the maintenance of competence and transformation. HGR accelerating rates of adaptive evolution is, however, only one of at least three non-exclusive mechanisms that operate synergistically to maintain competence for the uptake of exogenous DNA. The other three are; (1) the acquisition of templates for the repair of double stranded breaks in DNA [46],[47]; the uptake of nutrients and nucleotides [48],[49],[50],[51], and (3) episodic selection favoring transiently non-growing subpopulations of competent cells and rare transformants [31]. In accord with these three hypotheses, transformation (recombination) is a coincidental byproduct of competence.

As is the case with meiotic recombination in eukaryotes, accounting for the selective pressures responsible for original evolution of competence and transformation is more problematic than explaining their maintenance once they have evolved. Competence is a complex character that requires the coordinated activity of a large number of genes [15],[52],[53],[54],[55]. What are the selective pressures responsible for the evolution of these genes and coordinating their activity? Because recombination will only be favored when it is common, we postulate that HGR accelerating rates of adaptive evolution cannot account for the original evolution of natural competence and transformation. For the same reasons, we postulate that this is also the case for the episodic selection for competence. The DNA repair and food hypotheses have the virtues of selection operating at the level of an individual bacterium rather than populations and thereby allowing competence to be favored when it is rare, rather than only when it is common. On the other hand, these two hypotheses raise other issues about whether they can account for the original evolution of competence. For a recent critical consideration of these “other issues” we refer the reader to the Discussion in [31]. At this juncture, we accept the selection pressures responsible for the origins of competence and transformation in bacteria as a delicious, but yet-to-be solved evolutionary problem.

### A Caveat

In our simulations we have restricted the theater of evolution to single populations. A long-standing argument for the evolution of recombination is that higher rates of adaptive evolution provide an advantage to the collective, the group, rather than individuals [19],[29]. Populations that evolve more rapidly are more likely to prevail and survive longer than those with lower rates of adaptive evolution. In theory there are conditions where group- or interpopulation- level selection can lead to the evolution of characters that are at a disadvantage within populations [56],[57],[58],[59]. And, mechanisms of this type have been postulated to play a role in the evolution of recombination in bacteria [60]. While we prefer individual-level selection operating within populations on the grounds of parsimony, we can't rule out the possibility that competence and transformation evolved and is maintained by some form of group- level selection.
